# Translational control by cytoplasmic polyadenylation in *Xenopus* oocytes

**DOI:** 10.1016/j.bbagrm.2008.02.002

**Published:** 2008-04

**Authors:** Helois E. Radford, Hedda A. Meijer, Cornelia H. de Moor

**Affiliations:** School of Pharmacy, Centre for Biomolecular Sciences, University of Nottingham, University Park, Nottingham NG7 2UH, UK

**Keywords:** Cytoplasmic polyadenylation, Oocyte, Meiotic maturation, Translational control, Deadenylation

## Abstract

Elongation of the poly(A) tails of specific mRNAs in the cytoplasm is a crucial regulatory step in oogenesis and early development of many animal species. The best studied example is the regulation of translation by cytoplasmic polyadenylation elements (CPEs) in the 3′ untranslated region of mRNAs involved in *Xenopus* oocyte maturation. In this review we discuss the mechanism of translational control by the CPE binding protein (CPEB) in *Xenopus* oocytes as follows:

1. The cytoplasmic polyadenylation machinery such as CPEB, the subunits of cleavage and polyadenylation specificity factor (CPSF), symplekin, Gld-2 and poly(A) polymerase (PAP).
2. The signal transduction that leads to the activation of CPE-mediated polyadenylation during oocyte maturation, including the potential roles of kinases such as MAPK, Aurora A, CamKII, cdk1/Ringo and cdk1/cyclin B.
3. The role of deadenylation and translational repression, including the potential involvement of PARN, CCR4/NOT, maskin, pumilio, Xp54 (Ddx6, Rck), other P-body components and isoforms of the cap binding initiation factor eIF4E.

Finally we discuss some of the remaining questions regarding the mechanisms of translational regulation by cytoplasmic polyadenylation and give our view on where our knowledge is likely to be expanded in the near future.

## 1 Introduction

The regulation of translation of mRNAs by cytoplasmic elongation of the poly(A) tail was discovered some twenty years ago in the oocytes and early embryos of clams, worms, frogs and mice [1–6]. These maternal mRNAs are stored in the growing oocyte with a short poly(A) tail of 20 to 40 nucleotides and are translationally repressed (masked). Upon oocyte maturation or after fertilisation, the poly(A) tail of masked mRNAs is elongated to 80–250 residues and the mRNAs are translationally activated. A long poly(A) tail is thought to be stimulatory to translation through the binding of cytoplasmic poly(A) binding proteins, which recruit initiation factors and form a closed-loop complex through their association with the translation initiation factor eIF-4G [7]. Cytoplasmic polyadenylation is essential for the meiotic maturation of the oocyte in both *Xenopus* and mouse, as it mediates the translational activation of the mRNAs encoding c-Mos kinase and mitotic cyclins [8]. In addition, cytoplasmic polyadenylation has been implicated in the cell cycle, in cellular senescence and in the formation of memory through synaptic plasticity [9]. In this review we will discuss the current knowledge of the mechanism of translational control by cytoplasmic polyadenylation, with an emphasis on *Xenopus* oocyte maturation and CPEB, the cytoplasmic polyadenylation element binding protein. A review of the role of cytoplasmic polyadenylation in mammalian gametogenesis is provided elsewhere in this volume (Tadashi Baba).

## 2 mRNA elements and RNA binding proteins

In order for mRNAs to be singled out for cytoplasmic polyadenylation, they have to be recognised by the polyadenylation machinery. In oocytes and early embryos of the African clawed frog, *Xenopus*, cytoplasmic polyadenylation can be conferred by several discrete elements in the 3′ untranslated region (3′ UTR) of the mRNA which are recognised by specific RNA binding proteins. At least 4 different cytoplasmic polyadenylation elements have been identified in *Xenopus*. The C-rich cytoplasmic polyadenylation element and the U-rich embryonic cytoplasmic polyadenylation element normally mediate cytoplasmic polyadenylation in the zygote and early embryo [10,11]. The putative cytoplasmic polyadenylation factors binding to these elements are poly(rC) binding protein 2 (PCBP2) and Elav related protein A, the ortholog of HuR, respectively [10,12–14]. A polyadenylation element that functions early in oocyte maturation, the polyadenylation response element (PRE), was predicted computationally [15,16]. The RNA binding protein Musashi was shown to bind to some, but not all PREs and mutating Musashi or its known consensus binding site blocked cytoplasmic polyadenylation, indicating that it is likely to be a cytoplasmic polyadenylation factor [17]. These data suggest that the collection of cytoplasmic polyadenylation elements that have been called PREs may represent a mixture of Musashi binding sites and at least one more uncharacterised cytoplasmic polyadenylation element.

By far the best characterised cytoplasmic polyadenylation element is the CPE, which is required for the cytoplasmic polyadenylation of a number of mRNAs, including cyclin B1 mRNA, during oocyte maturation and during the embryonic cell cycle [18–20]. The consensus CPE is often described as U_5_AU, but variations such as U_4_AU (c-mos), and U_4–5_A_2_U (cyclin B1) have also been shown to be active [5,6,21,22]. The CPEs in the mRNA encoding mouse c-Mos are active in mature *Xenopus* oocytes, but only conform to U_5_A [23,24]. Strongly polyadenylating mRNAs, however, generally adhere to the U_5_A_1–2_U consensus. In addition, examination of the cases in the existing literature suggests that CPEs that mediate significant polyadenylation tend to be close to the polyadenylation signal, from downstream to overlapping and up to approximately 60 nt upstream [21,25–29].

The CPE binds cytoplasmic polyadenylation element binding protein (CPEB), an RNA binding protein containing two RNA recognition motifs and a zinc finger region, all of which are required for recognition of the CPE [21,30,31]. In addition to the fact that many mRNAs bearing a CPE undergo cytoplasmic polyadenylation, a wealth of evidence indicates that CPEB is required for cytoplasmic polyadenylation. Depletion of CPEB from an egg extract abolishes polyadenylation and injection of a CPEB antibody blocks it in oocytes and embryos [20,21,31,32]. N-terminal deletions and phosphorylation site mutants of CPEB act as dominant negative blockers of cytoplasmic polyadenylation [20,21,31,32]. In mice, knock out or knock down of Cpeb1 causes poly(A) tail changes in target mRNAs in early oogenesis and oocyte maturation [33,34]. CPEB is therefore the only well-established mRNA specificity factor for cytoplasmic polyadenylation in vertebrates.

In addition to an mRNA specific element, the nuclear poly(A) signal (AAUAAA or AUUAAA) is absolutely required for cytoplasmic polyadenylation [5,6,11,22,28]. The poly(A) signal is thought to recruit a variant of the cleavage and polyadenylation specificity factor (CPSF), a four subunit complex that binds this element and mediates polyadenylation in the nucleus [35,36]. The 160 kDa subunit is the RNA binding protein that recognises the poly(A) signal [37], while the 73 kDa subunit is the endonuclease that mediates the formation of the 3′ ends of all mRNAs [38–41]. The 100 kDa subunit resembles the 73 kDa subunit but does not appear to have nuclease activity and its precise function in 3′ end processing is unknown. The 30 kDa subunit is a zinc finger protein that has been reported to have some affinity for RNA and it potentially has endonuclease activity. It was recently shown to mediate interactions between CPSF and the body of RNA polymerase II [42–45]. An additional CPSF associated factor, Fip1, has been shown to mediate the interaction with poly(A) polymerase and have U-rich RNA binding activity [46]. It has probably been present in most of the previously utilised preparations of CPSF and is thought of as a fifth subunit by many in the field [40,46].

The 100 and 30 kDa CPSF subunits are present in the *Xenopus* oocyte cytoplasm, and the 160 kDa subunit has been inferred to be present from its binding activity, while the 73 kDa endonuclease is absent from the cytoplasmic CPSF complex [47]. It is unknown whether Fip1 is present in the oocyte cytoplasm. Immunodepletion of the 100 kDa subunit from egg extracts or expression of a viral protein that blocks nuclear polyadenylation by binding to the 30 kDa subunit also abolishes cytoplasmic polyadenylation, demonstrating that CPSF is required for this process [47]. In addition, CPEB is coimmunoprecipitated with the 100 kDa CPSF subunit from oocyte extracts (but not with the 73 kDa subunit) and it binds to the 160 kDa subunit in reticulocyte lysate [47,48]. Whether these interactions are direct or mediated by other proteins present in both oocyte extract and reticulocyte lysate is not yet clear. Curiously, the binding of CPSF to the poly(A) signal is stimulated by a CPE even in the absence of CPEB, a phenomenon that could be mediated by the RNA binding activity of the 30 kDa subunit or by Fip1 [46,49]. However, CPEB strongly stimulates polyadenylation of a CPE containing RNA in a pure reaction system containing nuclear CPSF and poly(A) polymerase [48]. It is therefore likely that CPEB improves the recruitment of CPSF to the mRNA by binding directly to both.

## 3 Symplekin

Symplekin is a protein found in nuclear complexes containing CPSF and other processing factors and is thought of as a scaffold protein involved in 3′ end RNA processing [50–52]. In yeast and plants, homologues of symplekin are required for proper cleavage and polyadenylation, and this is possibly also the case in vertebrates [53–55]. In vertebrate somatic cells symplekin is predominantly nuclear and in epithelial cells also localised at tight junctions [50,56]. In the *Xenopus* oocyte, however, symplekin is also found in the cytoplasm in complexes with the 100 kDa subunit of CPSF [50]. A symplekin antibody also precipitates CPEB from oocyte extracts, indicating that the protein is in cytoplasmic polyadenylation complexes [57]. Moreover, the symplekin antibody inhibits cytoplasmic polyadenylation in oocytes and extracts [57]. These data strongly suggest that symplekin is involved in cytoplasmic polyadenylation.

## 4 The cytoplasmic poly(A) polymerase

Initially, a form of one of the classical nuclear poly(A) polymerases was thought to be responsible for cytoplasmic polyadenylation too. *In vitro* reconstitution experiments indicated that the poly(A) polymerase (PAP) isolated from bovine thymus can mediate CPE and poly(A) signal dependent polyadenylation [47–49]. Cytoplasmic poly(A) polymerase species were detected in *Xenopus* oocytes and an isoform was cloned that was 87% identical to bovine PAP but lacked the nuclear localisation signal [58,59]. Three different polyclonal antibodies against this protein inhibited polyadenylation in egg extracts [58]. However, after these findings the work on the cytoplasmic poly(A) polymerase stagnated. No native complex containing CPEB and a PAP isoform was ever reported and studies on the regulation of PAP activity indicated that the enzyme is inactivated by phosphorylation during oocyte maturation, when cytoplasmic polyadenylation is actually induced [60].

In 2002, a *Caenorhabditis elegans* germline determinant called *gld-2* was cloned and shown to encode a cytoplasmic poly(A) polymerase that is recruited to certain mRNAs by an RNA binding protein [61]. The Gld-2 protein belongs to the same large family of DNA polymerase β nucleotidyl transferases, but only has limited additional homology to the classical poly(A) polymerases and it lacks the RNA binding domain. It soon transpired that this protein is widely conserved, having homologues in fission yeast and mammalians [62,63].

The *Xenopus* Gld-2 protein (xGld-2) coimmunoprecipitated very efficiently with symplekin in both mature and immature oocytes. In addition, tagged xGld-2 was shown to bind the 160 kDa CPSF subunit as well as CPEB synthesised in reticulocyte lysate [57]. This indicates that xGld-2 can form complexes with symplekin, CPSF and CPEB, although the direct interactions are as yet unclear. Gld-2 overexpressing oocytes and extracts supplemented with Gld-2 displayed an increase in their CPE-dependent polyadenylation and symplekin depleted extracts regained some polyadenylation activity if they were replenished with recombinant CPEB, symplekin and Gld-2 [57].

The less than optimal reconstitution of cytoplasmic polyadenylation by Gld-2 may indicate that cytoplasmic PAP or an unknown additional cytoplasmic polyadenylation factor is being depleted with symplekin. However, it is also very plausible that not all the supplemented proteins are completely active. Especially CPEB is notoriously difficult to obtain in a soluble form and isolation procedures usually include a denaturing step [30]. On the whole, the evidence seems to indicate that xGld-2 is an authentic cytoplasmic poly(A) polymerase in *Xenopus* oocytes, but involvement of the cytoplasmic PAP can as yet not be excluded. In *Drosophila*, PAP has been implicated in cytoplasmic polyadenylation in embryos [64], and although initial experiments implicated mouse Gld-2 in cytoplasmic polyadenylation in oocytes, polyadenylation appeared unimpaired in a knock out mouse [65,66]. The cytoplasmic polyadenylation mechanisms in mammalian germ cells are discussed in more detail by T. Baba elsewhere in this volume.

What part of the cytoplasmic polyadenylation complex recruits either PAP or Gld-2 is also still an open question. It is possible that CPEB replaces Fip1 in cytoplasmic polyadenylation, or that another specific recruitment factor exists for Gld-2. Also, the nuclear cleavage stimulation factor CstF77 has been reported to be present in the cytoplasmic polyadenylation complex in *Xenopus* oocytes, but appears to have an as yet undefined role in translational repression, rather than in cytoplasmic polyadenylation [67].

In nuclear polyadenylation, binding of the nuclear poly(A) binding protein, PABP2, is required to limit the length of the poly(A) tail. It has been suggested that such a function is required in cytoplasmic polyadenylation, as symplekin immunoprecipitates from polyadenylating egg extracts add abnormally long poly(A) tails [57]. It is unknown if the length of the poly(A) tail added is controlled in cytoplasmic polyadenylation, but it is worth noting in this context that the main poly(A) binding protein in oocytes is not the somatic cytoplasmic PABP, but an embryo specific protein, ePABP, which may have a specific function in this context [68,69].

Recently, it was shown that ePAB and somatic PABP both can bind to CPEB, and that this interaction is reduced by phosphorylation of CPEB during oocyte maturation [65,70]. The ePABP–CPEB interaction has been proposed to stimulate cytoplasmic polyadenylation and translational activation, as demonstrated by depletion experiments [65]. ePABP may therefore be a cytoplasmic polyadenylation factor. However, the data presented appear to show that symplekin is partially depleted with ePABP, which could explain the reduction in polyadenylation. The add-back experiment does unfortunately only show a minimal restoration of polyadenylation. Depletion of CPEB with ePABP is described for experiments described in the discussion section of this paper, adding to these concerns. In addition, because most panels in this paper do not show direct comparisons between CPE containing and CPE lacking RNA substrates, the intriguing effects of ePABP depletion could perhaps also be explained by a general effect of enhanced deadenylation and reduced translation of all polyadenylated mRNAs, which are predicted effects of low ePABP levels [68,69]. For the moment, it therefore appears prudent to reserve judgement on the function of the CPEB–ePABP interaction.

## 5 The activation of cytoplasmic polyadenylation

In *Xenopus* oocytes, meiotic maturation is induced by steroid hormones, probably through the action of both classical nuclear steroid receptors and a G protein coupled transmembrane receptor [71–73]. A rapid drop in cyclic AMP leads to the inactivation of protein kinase A, which is necessary for the induction of cytoplasmic polyadenylation. In recent years, progress has been made in various aspects of the activation mechanisms of cytoplasmic polyadenylation, although the picture is far from complete.

Tethering of mammalian Gld-2 to an mRNA using a fusion with an RNA binding domain and an mRNA with a binding site causes strong polyadenylation in immature *Xenopus* oocytes, indicating that the protein is constitutively active and only needs to be recruited to the mRNA to elongate the poly(A) tail [62]. However, as described above, Gld-2 is already associated with the polyadenylation factors in immature oocytes [57]. In addition, Gld-2 overexpression also leads to robust polyadenylation of CPE containing RNA in immature oocytes, which implies that the protein is probably inactivated by factors that are titrated by the overexpressed protein [57]. Since the action of Gld-2 requires recruitment to the RNA for activity, the titrated factor is unlikely to be CPEB itself. An intriguing possibility is that the protein depleted by Gld-2 is the cytoplasmic isoform of poly(A) ribonuclease (PARN). A 64 kDa catalytically active fragment of PARN has been found to coimmunoprecipitate with symplekin, CPEB and Gld-2 in immature but not in mature oocytes and binds to CPEB and Gld-2 synthesised in reticulocyte lysate [74]. The full length PARN is not abundantly found in this complex. The 64 kDa PARN isoform is thought to be responsible for the short poly(A) tails of CPE containing mRNAs, as a PARN antibody inhibited CPE-mediated deadenylation in immature oocytes [74]. In addition, catalytically inactive PARN can induce polyadenylation of CPE containing RNAs in immature oocytes and overexpression of wild type PARN represses the polyadenylation induced by Gld-2 overexpression [74]. These data indicate that PARN is the repressive factor depleted by Gld-2 injection and that it is the ejection of PARN from the polyadenylation complex that induces cytoplasmic polyadenylation [74]. There are two possibilities to explain the action of PARN on CPE containing mRNAs, either Gld-2 continuously adenylates while PARN takes these nucleotides off, or the presence of PARN blocks Gld-2 activity, for instance by preventing access to the 3′ end of the mRNA.

Since not all mRNAs are activated by cytoplasmic polyadenylation at the same time, the activation of the complexes must depend at least in part on mRNA specificity factors. In maturing *Xenopus* oocytes, cytoplasmic polyadenylation has been classed into 2 types, early (class I) and late (class II). Early polyadenylating mRNAs gain a poly(A) tail well before the activation of cdk1/cyclin B and the breakdown of the nuclear envelope (germinal vesicle breakdown, GVBD). Early polyadenylation is independent of the synthesis of the kinase c-Mos (which is itself encoded by a class I mRNA), while late polyadenylating mRNAs are dependent on c-Mos synthesis and the subsequent cdk1/cyclin B activation and gain their tails after GVBD [16,75,76]. The less well characterised polyadenylation response element (PRE, see above) and its binding factor Musashi have been shown to be responsible for some of the early polyadenylation events, despite the fact that these mRNAs also contain CPEs [15,17]. However, there is a clear correlation between the placement of the CPEs in an mRNA and the timing of its polyadenylation [15,77,78]. In addition, some late polyadenylating mRNAs also contain PREs/Musashi binding sites and the histone B4 mRNA, which contains both a PRE and a CPE, has been reported to be polyadenylated early, but has been shown to be Mos-dependent, indicating that the current classification in early and late perhaps oversimplifies the situation [15,17,75,78].

The evidence for which factor mediates early polyadenylation rests primarily on the use of dominant negative Musashi and CPEB mutants, which block translation and polyadenylation of c-Mos mRNA and GVBD [15,17,48]. Charlesworth et al. compared Musashi and CPEB mutants, and came to the conclusion that Musashi alone mediates the early polyadenylation, in contrast to earlier findings by Mendez et al., which implicated CPEB in the early polyadenylation [17,32]. The CPEB phosphorylation mutant used by Charlesworth et al. is a rather weak dominant negative (L.E. Hake, Boston, USA, unpublished observation), so it is possible that CPEB-dependent early polyadenylation is not completely repressed by it in all cases. Differences in expression levels and examination of endogenous or injected RNAs could also explain the discrepancies between the two studies. In addition, either or both dominant negative proteins could be titrating other cytoplasmic polyadenylation factors and inhibit polyadenylation on mRNAs to which they do not normally control, or they could fail to enter a specific mRNA–protein complex due to low exchange rates. Importantly, injection of a CPEB antibody prevented GVBD and gave strong repression of polyadenylation of injected early RNAs, including a Mos 3′ UTR construct, indicating that CPEB-mediated early polyadenylation exists [21]. In addition, interference with pathways that are known to affect CPEB phosphorylation and activation leads to a reduction in pre-GVBD c-Mos mRNA polyadenylation and a delay or block in oocyte maturation, including in those papers that claim that early polyadenylation is CPEB-independent [15,16,32,79,80]. However, there remains the possibility that the same signal transduction pathways ultimately activate Musashi and CPEB, which would make it impossible to distinguish between the effects of the two proteins in these experiments, with the possible exception of the CPEB antibody injection. With the limitations of the currently available evidence, it seems safest to assume that Musashi and CPEB both contribute to the polyadenylation of early mRNAs and that some mRNAs may be exclusively dependent on one or the other. Further work is required to resolve this issue definitively.

The induction of *Xenopus* oocyte maturation and early cytoplasmic polyadenylation by progesterone is dependent on a drop in cAMP levels and the resulting inhibition of protein kinase A [17,81]. Downstream of this event is the phosphorylation of CPEB on Ser174, which happens early in oogenesis. Although CPEB, CPSF and Gld-2 are already in a complex in immature oocytes, this phosphorylation appears to induce a stronger association of CPEB with CPSF and Gld-2 [32,48,82]. Importantly, Ser174 phosphorylation also induces the ejection of PARN from the polyadenylation complex [74]. Mutation of Ser174 to alanine results in a dominant negative protein that inhibits oocyte maturation and early polyadenylation partially or fully and late polyadenylation completely [15,32,83]. In addition, injection of a peptide containing the phosphorylation site delays GVBD considerably, suggesting that it is inhibiting an early polyadenylation event [32]. All evidence therefore indicates that phosphorylation of Ser174 is a crucial event in the activation of cytoplasmic polyadenylation.

There is some controversy surrounding the kinase mediating the early phosphorylation of CPEB on Ser174. Initially, it was demonstrated that Aurora A kinase (Eg2) can phosphorylate this site and that this requires the N-terminus of CPEB, in addition to the phosphorylation site itself [32]. This suggested that the N-terminally deleted CPEB may owe its dominant negative properties to the lack of a binding site for Aurora A. In addition, *in vitro* polyadenylation reactions containing recombinant CPEB, purified CPSF and nuclear PAP are strongly stimulated by the addition of Aurora A [48]. Glycogen synthase kinase 3 (GSK3) is thought to negatively regulate Aurora A by phosphorylation and this block appears to be relieved during oocyte maturation [83]. Consistent with a role in early oocyte maturation, GSK3 overexpression inhibited GVBD in *Xenopus* oocytes [83]. However, studies from three independent groups have failed to detect Aurora A activation early in oocyte maturation [79,84–86]. In addition, Aurora A inhibitors do not affect oocyte maturation or CPEB phosphorylation and depletion of Aurora A from oocyte extracts does not inhibit early CPEB phosphorylation either [79]. There is therefore sufficient evidence to throw serious doubt on the role of Aurora A in the activation of CPE-mediated cytoplasmic polyadenylation early in oogenesis. In mouse neurons, calmodulin dependent kinase II (CamKII) can phosphorylate the corresponding site on mouse CPEB, Thr171 [87]. CamKII plays a role in the induction of mouse oocyte maturation and is known to be present in *Xenopus* eggs, and could therefore be an alternative candidate kinase for mediating the early phosphorylation [88–91]. Because CamKII can be activated independently of calcium, the absence of a calcium flux in early oocyte maturation is not necessarily an obstacle for this hypothesis [92]. Evidence for a requirement of CamKII activity early in oocyte maturation would be necessary to make this more than speculation. However, Aurora A is very active later in oogenesis and it is undeniably capable of activating CPEB. It therefore appears likely that at the very least the maintenance of CPEB phosphorylation on Ser174 is carried out by this kinase.

Mitogen activated protein kinase (MAP kinase) has been implicated in the activation of early CPE-mediated, but not PRE-mediated, cytoplasmic polyadenylation [16,17,79,93]. An early, c-Mos independent activation of MAP kinase has been detected during oocyte maturation and CPEB is phosphorylated by MAP kinase on multiple sites [32,79,94]. MAP kinase does not phosphorylate Ser174, but has been suggested to either prime CPEB for Ser174 phosphorylation or to activate the Ser174 kinase [79]. As the early MAPK activation is dependent on protein synthesis, translational activation of another mRNA is probably required [94]. One potential candidate is RINGO/Speedy, a Cdk1 interacting and activating protein that is transiently expressed after progesterone treatment and required for oocyte maturation and CPEB phosphorylation [65,95–98].

A guanine nucleotide exchange factor for the Rho family of GTPases was found to interact with the N-terminal half of CPEB in a yeast 2 hybrid screen and called XGef [80]. This interaction was confirmed in oocytes and has been shown to be direct [80,99]. There appear to be 2 binding sites for CPEB on XGef [99]. Antibodies against XGef block oocyte maturation and prevent the polyadenylation and translation of c-Mos mRNA [80,99]. Conversely, overexpression of XGef accelerates oocyte maturation and c-Mos polyadenylation, independent of the production of c-Mos protein, indicating that it is upstream of this kinase in the signal cascade of meiotic maturation [80]. XGef enhances the early phosphorylation of CPEB and the DH domain associated with nucleotide exchange activity is required for its effects of on cytoplasmic polyadenylation [80,99]. In addition, a mutant of XGef that retains nucleotide exchange activity but has impaired binding to CPEB reduced early CPEB phosphorylation and delayed oocyte maturation [99]. However, a broad spectrum Rho GTPase inhibitor did not affect oocyte maturation or CPEB phosphorylation, indicating that the DH domain may not function to activate a Rho family GTPase, but is required in another capacity [79]. Strikingly, XGef immunoprecipitates were found to contain MAPK in both immature and mature oocytes and it therefore may be required to bring CPEB to the signalling complexes involved in its phosphorylation [79].

In another yeast 2 hybrid study, mouse CPEB was found to bind the small intracellular domain (ICD) of the transmembrane protein amyloid precursor like protein 1 (APLP1) and its relatives [100]. In *Xenopus* oocytes, Gld-2, the CPSF 100 kDa subunit and symplekin were predominantly associated with the plasma membrane in the same fractions as overexpressed APLP1 and immunoprecipitation of CPEB from these fractions indicates that it can associate with APLP1 [100]. Immunogold electron microscopy confirmed that CPEB and CPE containing RNA is associated with membranes. Overexpression of full length APLP1 induced some cytoplasmic polyadenylation in untreated oocytes and enhanced the effect of low concentrations of progesterone. The ICD alone had even stronger effects, indicating that the action of APLP1 is not dependent on membrane localisation. After treatment with low concentrations of progesterone, APLP1 stimulated CPEB phosphorylation on Ser174 [100]. While the association with amyloid precursor proteins may have great significance for the role of CPEB and cytoplasmic polyadenylation in neurons, it is as yet unclear whether APLP1 or its relatives are required for cytoplasmic polyadenylation in oocytes and whether it mediates the membrane association of the cytoplasmic polyadenylation complexes.

Late cytoplasmic polyadenylation is required for progression from meiosis I to meiosis II during oocyte maturation [101]. mRNAs that undergo late polyadenylation often have a CPE overlapping with their poly(A) signal, e.g. UUUUAAUAAA [15,77,78]. The late polyadenylation during oocyte maturation is dependent on the activation of the mitotic kinase cdk1 and its regulatory cyclin subunits [16,77,78,93]. Cdk1 does phosphorylate CPEB leading to degradation of most of the oocyte CPEB via ubiquitin-mediated degradation [101–103]. This degradation is required for late polyadenylation to occur. Remarkably, symplekin bound CPEB appears to represent the stable fraction of CPEB, as symplekin immunoprecipitates do not show a difference in CPEB content between immature and mature oocytes [57]. This implicates that CPEB in polyadenylation complexes is stable during oocyte maturation, consistent with the polyadenylation activity observed. A model for late cytoplasmic polyadenylation can be devised in which the abundant ‘free’ CPEB (i.e. CPEB in a different complex than symplekin, CPSF and the poly(A) polymerase) binds to the CPE overlapping the poly(A) signal in late polyadenylating mRNAs and prevents the recruitment of CPSF and symplekin by the polyadenylation signal early in oocyte maturation. After cdk1 activation, most free CPEB is depleted and a complex containing CPEB, symplekin and CPSF binds both the CPE and the poly(A) signal [101].

**Fig. 1:**
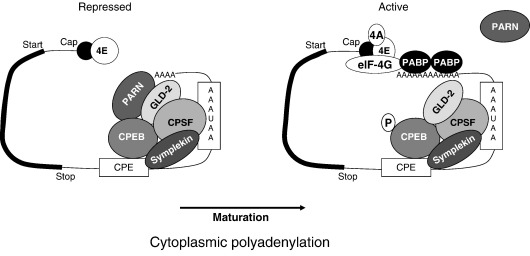
The activation of CPE-mediated cytoplasmic polyadenylation. In immature oocytes, CPE containing mRNAs already contain all the proteins necessary for polyadenylation as well as the deadenylase PARN. During oocyte maturation, phosphorylation of CPEB causes a rearrangement of the complex that leads to the ejection of PARN and the activation of the poly(A) polymerase Gld-2. 4E: eIF4E, 4A: eIF4A. For further description of this process, see the text.

**Fig. 2:**
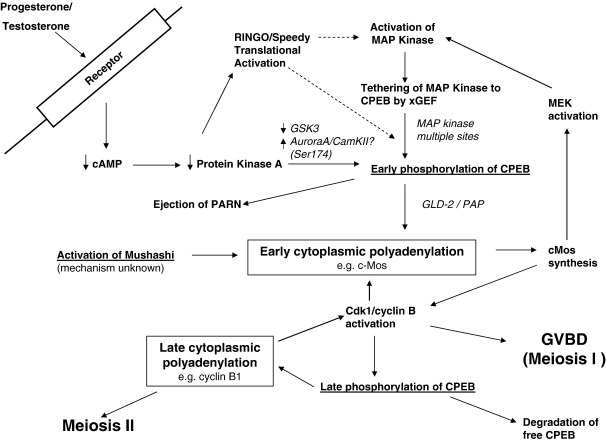
The signal transduction cascade leading to cytoplasmic polyadenylation during oocyte maturation. Schematic representation of the key signalling events in oocyte maturation and the role of cytoplasmic polyadenylation in this process. For a detailed description, see the text.

A current view of CPE-mediated cytoplasmic polyadenylation in oocyte maturation is depicted in Figs. 1 and 2 and can be summarised as follows:

1. The activation of CPE-mediated cytoplasmic polyadenylation during progesterone induced oocyte maturation is induced by a drop in protein kinase A activity and requires an early translation event, perhaps translation of RINGO/Speedy.
2. This induces the early activation of MAP kinase, which is associated with the polyadenylation complex through XGef and phosphorylates CPEB on multiple sites, but not on Ser174.
3. CPEB is phosphorylated on Ser174 by an as yet not fully confirmed kinase, possibly Aurora A or CamKII, which is required for the induction of CPE-mediated polyadenylation and causes an increase in the binding between Gld-2, CPSF and CPEB, causing the ejection of PARN from the complex and allowing Gld-2 to elongate the poly(A) tail of the mRNA.
4. After GVBD, CPEB is phosphorylated by cdk1 and the free CPEB is mostly degraded, allowing the CPEB in polyadenylation complexes (now probably phosphorylated on Ser174 by Aurora A) to activate cytoplasmic polyadenylation on mRNAs which contain a CPE overlapping with the poly(A) signal.

A surprising number of proteins involved in the activation of polyadenylation are themselves upregulated by this process. This should create positive feedback loops which amplify the signal and contribute to the progression of meiotic maturation. As discussed above, the mRNA for the serine/threonine kinase c-Mos is one of the early targets of cytoplasmic polyadenylation [16,75,76]. It is an activator of the MAPK kinase MEK, and its synthesis leads to further activation of MAP kinase, activation of cdk1 and phosphorylation of CPEB [71]. The mitotic cyclins, activators of cdk1, are induced by cytoplasmic polyadenylation, as is Aurora A [19,26]. Similarly, CamKII is regulated by cytoplasmic polyadenylation in neurons [104]. Finally, Gld-2 targets its own mRNA, which contains CPEs in its 3′ UTR[105]. A system with so much positive feedback will require strong brakes, and it appears that at least in some cases this is provided by deadenylation factors, as discussed below.

## 6 Deadenylation and translational repression

The maternal mRNAs that are stored in the oocyte in an untranslated state have short poly(A) tails, but have been reported to have normal poly(A) tail addition in the nucleus in both mouse and frog [74,106]. In both organisms, the deadenylation of RNA injected into the cytoplasm requires CPEs, indicating that the CPEB–PARN complex, discussed above, mediates this process [74,106]. However, RNA substrates containing CPEs alone have not been tested in these experiments and it is therefore not clear if a CPE is sufficient for deadenylation or if other sequence elements are required. In fact almost all CPE containing mRNAs have putative binding sites for other deadenylation factors.

Firstly, the deadenylation factor Pumilio binds directly to CPEB, and therefore could contribute to the deadenylation of all CPE containing mRNAs [107,108]. Many CPE containing mRNAs also contain Pumilio binding sites, including cyclin B1 and Gld-2 [97,107–109], indicating that the recruitment of Pumilio maybe both by protein–protein and RNA–protein associations. Pumilio is a member of a highly conserved family of RNA binding proteins called the Puf family. Members of this family can mediate translational repression and mRNA destabilisation in organisms from yeast to vertebrates [110,111]. In yeast, the Puf family protein Mpt5 interacts with the conserved deadenylase complex CCR4–Pop2–Not, by binding to Pop2/Caf1 [112–114]. This association was also confirmed for the human and worm proteins [113]. In addition, Pumilio is known to aid the recruitment of the translational repressor Nanos, both in fly and frog [107,115]. Nanos can recruit the CCR4–Pop2–Not complex by binding to Not4 and contributes to the translational repression of fly cyclin B [116]. So far, the role of the CCR4–Pop2–Not complex in poly(A) tail metabolism has not been studied in *Xenopus* oocytes and embryos, but the conservation of both the recruitment proteins and one of the target mRNAs (cyclin B) seems to indicate that it is very likely to play a role.

A subset of mRNAs that are activated by CPE-mediated cytoplasmic polyadenylation during oocyte maturation loose their poly(A) tails after fertilisation [19,117,118]. In most cases, this is mediated by an embryonic deadenylation element (EDEN) in the 3′ UTR of the mRNA, which binds the deadenylation factor EDEN-BP [119]. Aurora A, cyclin B1 and c-Mos encoding mRNAs all bind EDEN-BP and the 3′ UTR of Gld-2 mRNA contains good consensus binding sites, making EDEN-BP a good candidate repressor of the cytoplasmic polyadenylation-mediated positive feedback loops during early embryogenesis [105,119–121]. The mammalian ortholog of EDEN-BP, CUGBP1, can recruit PARN [122]. A similar association of EDEN-BP and PARN in the *Xenopus* embryo could explain its role in deadenylation, but so far this complex has not been reported. It can therefore not be excluded that EDEN-BP mediates its deadenylation activity in *Xenopus* embryos through another deadenylase, for instance the CCR4–Pop2–Not complex. No direct contacts between CPEB containing and EDEN containing complexes has been described so far. Because CPEs, EDEN sequences, Musashi and Pumilio binding sites often occur in the same 3′ UTR, their associated complexes can be expected to compete for the end of the mRNA to mediate deadenylation or polyadenylation.

**Fig. 3:**
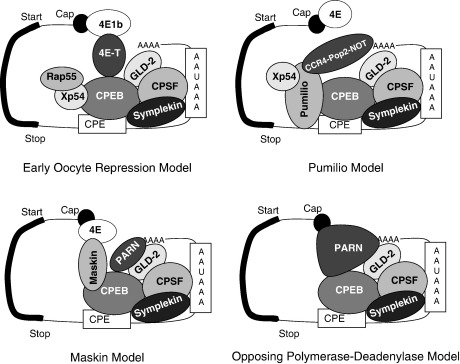
Four models of translation repression by CPEs. Schematic representation of some of the proposed translational repression mechanisms for CPE containing mRNAs. For a detailed description, see the text.

Although a short poly(A) tail will lead to inefficient translation of an mRNA, it is not sufficient for complete translational repression, as illustrated by the partial polysomal association of histone B4 mRNA and the accumulation of B4 protein in *Xenopus* oocytes [78,123,124]. Many of the other stored maternal mRNAs have to be strongly translationally repressed to enable the subsequent stages of growth, maturation and fertilisation of the oocyte [71]. CPEs, Musashi and Pumilio binding sites can repress translation of specific mRNAs, often in the absence of a poly(A) tail or in the absence of deadenylation, indicating that they have an additional function in blocking translation [75,105,107–109,125–127]. Several detailed models exist to explain the translational repression by CPEs, some of which are presented in Fig. 3.

In full grown oocytes, a CPEB binding factor called maskin was found to associate with the cap binding initiation factor eIF4E in a manner that should preclude recruitment of eIF4G and thus inhibit translation [128]. Maskin is a homologue of the transforming acidic coiled-coil domain protein 3 (TACC3) and these proteins play a vital role in the formation of the mitotic spindle in multiple organisms [129–134]. The binding between maskin and eIF4E is weak but detectable and this association is abrogated by binding of PABP to the poly(A) tail elongation and by phosphorylation of maskin in both oocytes and embryos [129,135–137]. As the eIF4E binding site in maskin appears not to be present in other organisms, it is not clear how widespread this regulation is [138]. A more conserved CPEB associated eIF4E binding protein is the neuronal protein neuroguidin, which has been proposed to function in a similar manner to maskin in repressing translation in the nervous system [139].

The deadenylase PARN is a well characterised cap binding protein and a 5′ cap structure is required for deadenylation by PARN [140–142]. CPEB can recruit PARN to the mRNA, but deadenylation still requires a cap structure [74]. This leads to a conflict between the maskin model and the opposing polymerase-deadenylase model [74,128]. Either the maskin model is true and eIF4E/maskin is stably bound to the cap and PARN is not continuously active, or the opposing polymerase-deadenylase model is correct and CPEB bound PARN is stably bound to the cap and continuously active, excluding eIF4E from the cap. In fact, PARN could very well mediate both the deadenylation and the translational repression of mRNAs to which it is recruited by excluding eIF4E from the mRNA [120].

Both maskin and PARN are absent in early oocytes, so other repression mechanisms must exist to mediate translational repression in these cells. Recently, a large CPEB containing RNP complex was found in early oocytes [143]. This complex contains several proteins found in P-bodies and related RNP granules that have been implicated in both translational control and mRNA degradation [144]. In mammalian cells, CPEB can also be found in large RNP granules including P-bodies [145]. The early oocyte CPEB complex contains the eIF4E variant eIF4E1b, the eIF4E binding protein 4E-T, the RNA helicase Xp54 (Ddx6, Rck, Me31B, Dhh1) and the P-body components p100 (Pat1) and Rap55 (Scd6, CAR-1). It does not contain PARN, maskin or the canonical cap binding initiation factor eIF4E1a. Rap55 and Xp54 can mediate translational repression in *Xenopus* oocytes when tethered to an mRNA [146–148]. In addition, the yeast homologues of both proteins, as well as of p100, are involved in general repression of translation [149]. Strikingly, the yeast Pumilio homologue Mpt5 recruits Dhh1, a homologue of the Xp54 RNA helicase, indicating that Pumilio-mediated translational repression may involve a similar complex [113]. The mechanism of this highly conserved type of translational repression is not entirely clear, but it is likely to involve assembly of large RNP particles that exclude translation initiation factors and/or ribosomes. In addition, eIF4E1b was shown to be defective in eIF4G binding and to bind 4E-T independently of the canonical eIF4E binding site. This implies that the mRNAs in this complex are translationally repressed through sequestration of the cap by eIF4E1b, which would exclude eIF4E1a/eIF4G from the mRNA. Indeed, tethering of 4E-T causes translational repression and injection of an eIF41b antibody enhances oocyte maturation [143]. It is as yet unclear which proteins in the early complex bind directly to CPEB and therefore are likely to confer specific repression of CPE containing mRNAs. As eIF4E1b is also the predominant CPEB associated eIF4E in fully grown oocytes, this protein could in principle convey CPE-mediated translational repression throughout oogenesis [67,143].

At present it is difficult to choose amongst the multitude of models for translational repression by CPEs. Some of the complexes detected by pulldown and immunoprecipitation may not contain the majority of the repressed mRNA, even though presence of some mRNA was demonstrated by RT-PCR. Alternatively, it may well be that every one of these models is correct at a particular stage of oogenesis or embryogenesis or that the repression is different for specific mRNAs, depending on binding sites for other proteins such as Pumilio and EDEN-BP. An attractive option is that sequestration to P-body like large complexes is the result of translational repression and acts as an enhancer of translational repression, while smaller, more mRNA specific translational repression complexes are formed during the movement of mRNAs in and out of P-bodies. To distinguish between these models, it will be important to characterise the cap binding proteins and P-body components present on specific mRNAs at different developmental stages.

## 7 Cytoplasmic polyadenylation and translational activation

The simplest explanation for why cytoplasmic polyadenylation leads to translational activation is that the poly(A) tail recruits translation initiation factors through its association with PABP and that these mediate release of the mRNA from the repression complexes, either by exchanging the cap binding complex for eIF4E1a/eIF4G or by somehow extracting the mRNA from a P-body-like RNP complex, a function which has been ascribed to PABP in yeast [150]. In support of this hypothesis, the translational repression of the histone B4 and cyclin B1 3′ UTRs on mRNAs injected into *Xenopus* oocytes can be abolished by the addition of a poly(A) tail, at least until that tail is deadenylated [29,74]. However other mRNAs (G10, Cl2) appear to be still repressed even if a long poly(A) tail is added and only become translated if the mRNA is actively polyadenylated in the maturing oocyte [6,13]. In a seminal experiment, Barkoff et al. cleaved the endogenous c-Mos mRNA with an oligonucleotide, removing the polyadenylation signal and CPE. As expected, this abrogated c-Mos mRNA polyadenylation and translation in oocytes exposed to progesterone. A ‘prosthetic’ poly(A) tail was tethered to the remaining 3′ UTR using basepairing. This did not lead to c-Mos synthesis in unstimulated oocytes, but restored the synthesis of c-Mos in response to progesterone [76]. This suggests that, at least for the translation of c-Mos mRNA, both the presence of a poly(A) tail and an additional, polyadenylation independent, modification of the mRNP are required for translational activation.

## 8 Discussion

Since the discovery of CPEB ten years ago, our knowledge of the mechanisms of CPE-mediated cytoplasmic polyadenylation has made great strides, especially in *Xenopus* oocytes. However, there are still quite a few questions that need to be resolved, for instance:

1. A more systematic investigation of the consensus CPE sequence and its maximum distance to the poly(A) signal would enable a more reliable bioinformatic prediction of the targets of CPE-mediated cytoplasmic polyadenylation. Are all mRNAs containing these sequences polyadenylated during oocyte maturation?
2. The relative roles of Gld-2 and PAP in cytoplasmic polyadenylation need to be further evaluated. Can they substitute for each other? What are their contact points in the cytoplasmic polyadenylation complex? A cytoplasmic polyadenylation system reconstituted from pure components would be ideal to resolve these questions.
3. The kinase(s) responsible for the early activating phosphorylation on Ser174 of CPEB should be identified unequivocally. If Aurora A is responsible, why do inhibitors of its activity not block oocyte maturation and can no early activation of this kinase be detected? Do inhibitors of CamKII affect early polyadenylation? What is the signal transduction cascade leading to induction of the activating kinase?
4. What are the relative roles of Musashi and CPEB in the polyadenylation of c-Mos mRNA and other substrates? How do dominant negative mutants of CPEB and Musashi achieve their repressive functions? May there be off target effects through titration of common polyadenylation factors or signal transduction machinery?
5. What are the direct interaction between proteins and RNA elements in the cytoplasmic polyadenylation complex? Much of the work discussed here has been performed either in oocyte or reticulocyte lysates, where other interacting proteins may be present. It appears necessary that these interactions are mapped in detail *in vitro* or in a 2 hybrid system, as this could lead to predictions for the functions of dominant negative mutants and potentially clarify how different complexes are assembled.
6. Are the complexes on cytoplasmic polyadenylation sequences other than the CPE similar to the CPEB associated complexes? For instance, do Gld2 or PAP associate with the RNA binding proteins that recognise them?
7. Which models for translational repression and deadenylation of CPE containing mRNAs are correct in what stage of oogenesis? Again, a more intimate knowledge of the direct interactions involved in assembling the repression complexes is likely to yield important new investigative tools for resolving this question. In addition, the study of mRNP complexes assembled in vivo could yield some more conclusive answers.
8. Are the polyadenylation and translation repression complexes identical for each CPE containing mRNA or are there mRNA specific differences in the complexes? Immunoprecipitation followed by RT-PCR and RNA affinity chromatography can partially answer this question, but affinity purification methods that target specific mRNPs are likely to be crucial to obtain a full answer.

Some of the controversies discussed here are a result of the experimental restrictions of the *Xenopus* oocyte and egg systems. These systems are very good for biochemical assays both in injected oocytes and egg extracts and they provide ample material for purification and identification. However, the high levels of stable maternal proteins make clean knock down or knock out experiments impossible in most cases, which has led to a heavy reliance on dominant negative approaches. To resolve some of the outstanding issues we are therefore likely to have to look to vertebrate genetic systems such as mouse and/or to develop tissue culture based cytoplasmic polyadenylation systems that are amenable to siRNA knock down, for instance using neuronal cells or cells synchronised in the cell cycle.

The advent of high throughput screens will strongly impact the field of cytoplasmic polyadenylation in the near future as the results of RNP immunoprecipitation microarray experiments and poly(A) tail profiling studies will increase our knowledge of the range of mRNA substrates [9,151–153]. Such screens may also identify new cytoplasmic polyadenylation elements as well as novel cell types and biological processes in which this form of regulation is involved. Only one cytoplasmic polyadenylation element and its binding protein have so far been studied in detail, predominantly in oocyte maturation. As outlined above, a large number of important questions are still unresolved, even in this one system. With at least three other cytoplasmic polyadenylation sequences in existence in oocytes alone and the evidence that cytoplasmic polyadenylation also is involved in neuronal events and the mitotic cell cycle [9], there appears ample scope for expansion of the field in the near future.
